# RANK-C Expression Sensitizes ER-Negative, EGFR-Positive Breast Cancer Cells to EGFR-Tyrosine Kinase Inhibitors (TKIs)

**DOI:** 10.3390/genes12111686

**Published:** 2021-10-23

**Authors:** Chaido Sirinian, Anastasios D. Papanastasiou, Soren E. Degn, Theodora Frantzi, Christos Aronis, Dimitrios Chaniotis, Thomas Makatsoris, Angelos Koutras, Haralabos P. Kalofonos

**Affiliations:** 1 Molecular Oncology Laboratory, Division of Oncology, Department of Medicine, University of Patras, 26504 Patras, Greece; theodorafrantzi@gmail.com (T.F.); chrisaronis@upnet.gr (C.A.); tmakatsoris@upatras.gr (T.M.); angkoutr@otenet.gr (A.K.); kalofonos@upatras.gr (H.P.K.); 2 Department of Biomedical Sciences, University of West Attica, 12243 Athens, Greece; apapanasta@uniwa.gr (A.D.P.); dchaniotis@uniwa.gr (D.C.); 3 Department of Biomedicine, Aarhus University, DK-8000 Aarhus C, Denmark; sdegn@biomed.au.dk

**Keywords:** breast cancer, ER-negative, EGFR, TKIs, RANK-c

## Abstract

Background: We have previously shown that overexpression of RANK-c in ER-negative breast cancer cell lines attenuates aggressive properties of cancer cells, partially through a RANK-c/EGFR interaction. EGFR inhibition through TKIs in breast cancer has been tested in triple-negative disease settings with limited clinical benefit for patients. Here we test if expression of RANK-c in ER-negative breast cancer cells in conjunction with treatment with TK inhibitors (erlotinib or gefitinib) can affect survival and colony-forming capacity of cancer cells. Methods: Stably expressing MDA-MB-231-RANK-c and SKBR3-RANK-c cells were employed to test proliferation and colony formation in the presence of TKIs. In addition, Western blot analysis was performed to dissect EGFR related signaling cascades upon TK inhibition in the presence of RANK-c. Results: Interestingly the two RANK-c expressing, ER-negative cells lines presented with a distinct phenotype concerning TKI sensitivity upon treatment. MDA-MB-231-RANK-c cells had a higher sensitivity upon gefitinib treatment, while erlotinib decreased the proliferation rate of SKBR3-RANK-c cells. Further, colony formation assays for MDA-MB-231-RANK-c cells showed a decrease in the number and size of colonies developed in the presence of erlotinib. In addition, RANK-c seems to alter signaling through EGFR after TKI treatment in a cell type-specific manner. Conclusions: Our results indicate that ER-negative breast cancer cells that express RANK-c alter their sensitivity profile against tyrosine kinase inhibitors (erlotinib and gefitinib) in a cell type-specific and culture substrate-dependent manner.

## 1. Introduction

Breast cancer is a leading cause of death in women and almost one out of 8 will develop a breast malignancy through their lifetime [1]. Concerning the biology of breast cancer, there are two distinct biological entities in relevance to the expression of hormone receptors. More specifically estrogen receptor (ER) expression divides malignant breast tumors in ER-positive and ER-negative disease groups and these two entities present with fundamentally different biology, clinical course, and response to therapy [2]. Regarding therapeutic choices and outcomes, ER-negative breast cancer presents limited options other than chemo- and/or radiation therapy while patient outcomes upon treatment are poor compared to treatment of ER-positive disease [3].

ER-negative and especially triple-negative disease, that is estrogen receptor-, progesterone receptor- and HER2-negative breast cancer (ER^−^, PR^−^, HER2^−^), present with a lack of targeted treatment options and worse clinical outcomes [4,5]. The ER-negative cluster includes a HER2-positive group, and the groups of triple-negative (TN) and basal-like cancers that partially overlap each other. The basal-like group comprises cancers that lack hormone receptor expression and HER2 expression, thus can be partially included in the TN subgroup, and they are additionally characterized by the expression of genes related to the basal/myoepithelial mammary cell phenotype, such as CK 5/6, laminin, c-KIT, α6 integrin, fatty acid-binding protein 7, P-cadherin, epidermal growth factor receptor (EGFR), and NF-κB [1,3,5].

EGFR mutation or/and deregulated expression is a hallmark of multiple human malignancies, though not common in breast cancer except in the case of TN and Basal-like cancers [6]. EGF receptor can be targeted by multiple tyrosine kinase inhibitors (TKIs) such as erlotinib and gefitinib but also by monoclonal antibodies such as cetuximab and panitumumab [7]. Both targeted pharmaceutical approaches have significant clinical results in lung and colon cancer. However, until today, targeting EGFR in breast cancer has produced poor therapeutic results indicating possibly the need for better predictive biomarkers relevant to the response to anti-EGFR therapy in breast cancer [8].

We have previously identified RANK receptor isoform, RANK-c, as an inhibitor of ER-negative breast cancer cell aggressiveness both in vivo and in vitro [9]. Through a TRAF2 and EGFR interacting complex, RANK-c alters NF-κB activation and inhibits breast cancer migration, invasion and metastasis. In addition, RANK-c expression in ER-negative cell lines alters downstream EGF-related pathways such as MAPK/ERK and AKT [10]. With the above in mind, in this work we sought to test the response of ER-negative, RANK-c expressing breast cancer cells under EGFR TKI treatment in respect to cell proliferation, colony formation and downstream EGF-related signaling.

## 2. Materials and Methods

### 2.1. Cell Culture, Plasmids and Transfection

MDA-MB-231 and SKBR3 cell lines (obtained from ATCC) were cultured as reported previously [10]. Generation of stable cell lines was performed as described previously by electroporation using the Amaxa^®^ cell line nucleofector^®^ kits (LONZA, Walkersville, MD, USA). Plasmids employed for the production of stable cell lines were: pcDNA3.1/Hygro-RANK-c and empty vector (Invitrogen, Carlsbad, CA, USA) [10].

### 2.2. Immunoblotting

Cells were lysed in 1% NP-40 lysis buffer (150 mM NaCl, 20 mM HEPES, 0.5 mM EDTA, 1 mM Na_3_VO_4_, proteinase inhibitor cocktail (Calbiochem). Total cell extracts concentration was determined using Coomassie Brilliant Blue staining (Sigma-Aldrich) and resolved in 10% acrylamide gels and transferred onto PVDF membrane (Millipore, MA, USA) before immunoblotting with the appropriate antibodies overnight at 4 °C. Primary antibody incubation was followed by incubation with an HRP-conjugated secondary antibody (anti-goat, anti-rabbit; Santa Cruz Biotechnology Inc., Santa Cruz, CA, USA and anti-mouse; CST, Danvers, MA, USA).

### 2.3. Antibodies and Reagents

The antibodies employed in this study were: EGFR (E235, 04-338; 1:4000 Millipore, MA, USA), pEGFR-Y845 (AF-3394; 1:100, R&D systems), pEGFR-Y1173 (04-341; 1:1000, Millipore), pAKT-S473 (4060; 1:2000, CST), ERK1/2 (9102; 1:1000, CST), pERK1/2-T202/Y204 (4376; 1:1000, CST), pSRC-Y416 (2101; 1:1000, CST). The tyrosine kinase inhibitors erlotinib (Traceva) and gefitinib (Iressa) were kindly provided by Roche and AstraZeneca, respectively.

### 2.4. Colony Formation Assay

Cells were suspended in 1 mL of 10% FBS DMEM medium containing 0.5% agarose and plated on a semisolid medium (DMEM with 10% FBS and 0.7% agarose) in 12-well plates (2 × 10^4^ cells/well). Cells were then placed in a 37 °C and 5% CO2 incubator. The next day, TKIs were added to the supernatant growth medium and changed every two days. The IC50 values for each drug were calculated from a log ([drug]) vs. normalized response curve fit in Excel. Colonies of cells were counted after 12 days of incubation with drugs. Images were obtained using an inverted microscope (Axiovert 40 CFL, AxioCam ERc, Zeiss). All experiments were done in triplicates. Cell colony area was calculated with ROI Manager on Image J software.

### 2.5. Proliferation Assay and Cell Counting

The 3-(4,5-dimethylthiazol-2-yl)-2,5-dimethyltetrazolium bromide (MTT) assay and manual cell counting were performed in order to determine whether erlotinib or gefitinib affects the proliferation of RANK-c expressing MDA-MB-231 and SKBR3 cells. RANK-c expressing cells and the controls were plated at a density of 2 × 10^4^ cells per well in a 24-well plate. Cells were grown in working medium for 24 h followed by an additional 48 h-incubation with erlotinib or gefitinib at the appropriate concentration determined by the IC50 value as described above (Section 2.4). MTT stock (5 mg/mL in 1× PBS) at a volume equal to 1/10 of the medium was added to each well and incubated at 37 °C for 2 h. The medium was removed and 100 μL acidified isopropanol was added to each well. The solution was transferred to 96-well plates and immediately read on a microplate reader (Tecan, Sunrise, Magellan 2) at a wavelength of 570 nm. All experiments were done in triplicates.

### 2.6. Statistical Analysis

*T*-test was employed to compare means of colony area between groups. All results are expressed as mean ± SD from at least three independent experiments. All data were analyzed with the SPSS program (SPSS^®^ release 15.0, Chicago, IL, USA) and presented as mean ± SD. Any *p*-value less than 0.05 was considered statistically significant.

## 3. Results

### 3.1. RANK-C Expression in ER-Negative Breast Cancer Samples Affects ERBB Phosphorylation Status

**Figure 1:**
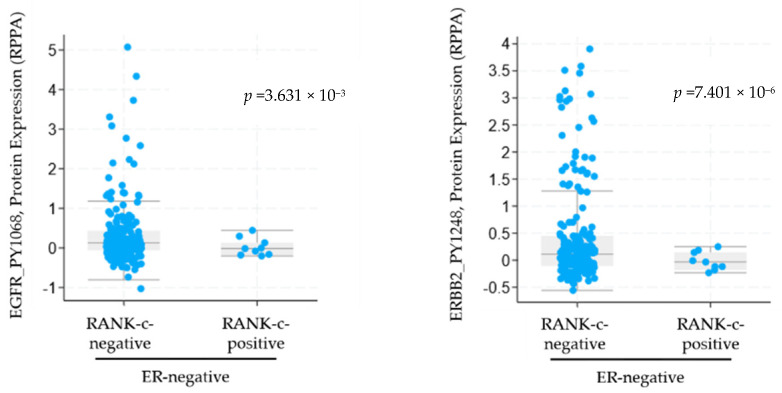
Analysis through the cBioPortal (cbioportal.org) for ER-negative breast cancer patients (*n* = 238) for the phosphorylation levels of EGFR and ERRB2 (HER2) depending on RANK-c expression. Phosphorylation of EGFR at tyrosine 1068 (left panel) and ERBB2 (HER2) at tyrosine 1248 is reduced in ER-negative samples with RANK-c expression.

In our previous work, we showed that RANK-c interacts with EGFR receptor in breast cancer cells affecting EGFR phosphorylation status and downstream signaling [10]. In order to compare protein phosphorylation between ER-negative patients that either expresses RANK-c or they are RANK-c-negative we compiled and analyzed through publicly available datasets (TCGA Breast Invasive Carcinoma. Source data from GDAC Firehose) two breast cancer patient cohorts [10,11,12]. We identified 12 patients as RANK-c-positive, ER-negative and 226 patients as RANK-c-negative, ER-negative with available data and employed cBioPortal to compare protein and phosphorylation levels of key genes in between groups [12]. Our analyses identified a statistically significant reduced phosphorylation at both EGFR^PY1068^ (*p* = 3.631 × 10^−3^) and ERBB2^PY1248^ (*p* = 7.401 × 10^−6^) in ER-negative, RANK-c-positive group of patients compared to the RANK-c-negative group (Figure 1). These data indicate that RANK-c expression may affect phosphorylation status of ERBB family members (EGFR, HER2) in ER-negative breast cancer patients, with possible implications in downstream signaling, cellular functions and response to targeted therapies.

### 3.2. EGFR Tyrosine Kinase Inhibitors Affects Heterogeneously ER-Negative, RANK-C Expressing Breast Cancer Cell Lines

In order to test, if the previously identified EGFR/RANK-c interaction [10] and the reduced phosphorylation encountered in ER-negative, RANK-c positive breast cancer samples (Figure 1) has an impact in the response to EGFR-TKI treatment of ER-negative breast cancer cells, we employed MDA-MB-231 and SKBR3 cells stably expressing RANK-c. Both cell lines are of the ER-negative subgroup and express high levels of EGFR and in the case of SKBR3 there is also an increased expression of HER2 (ERBB2).

**Figure 2:**
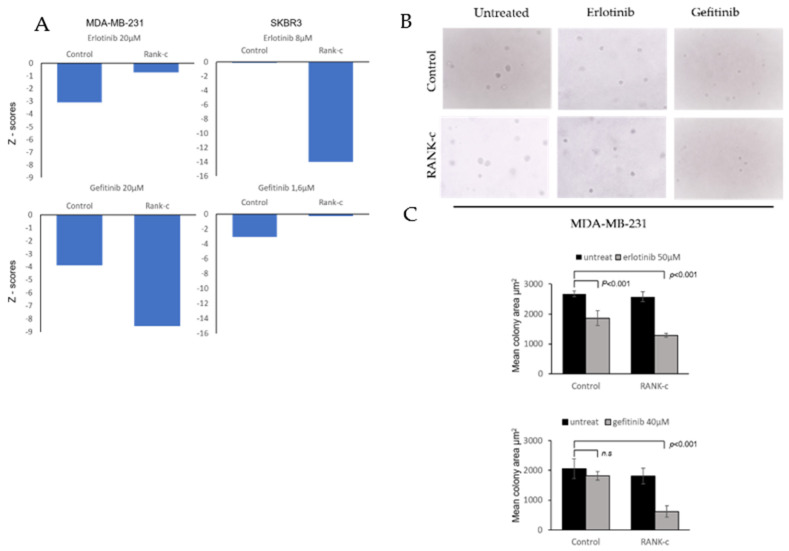
(**A**). Breast cancer cell lines MDA-MB-231 and SKBR3 were treated with the indicated TKI and quantified through an MTT assay after 72- and 48-h time period, respectively. Results presented in Z-scores. (**B**). Colony formation assay on soft agar for MDA-MB-231 cells treated with the indicated TKI (Erlotininb 50 μM and Gefitinib 40 μM), showing colonies of reduced size when RANK-c expression was combined with a TKI. (**C**). Quantification of mean colony area in soft agar from 2B through the ImageJ software. Z-score higher or lower than ±2 is statistically significant with a *p*-value < 0.05.

By employing a proliferation assay (MTT) and manual cell counting, MDA-MB-231-RANK-c and SKBR3-RANK-c cells were differentially affected when treated with the widely used EGFR tyrosine kinase inhibitors, erlotinib and gefitinib. Interestingly, gefitinib, but not erlotinib, after 72 h of treatment reduced the proliferation of MD-MB-231-RANK-c cells in comparison to control cells, while on the other hand, SKBR3-RANK-c cells were significantly more sensitive to a 48-h treatment with erlotinib (Figure 2A). To further expand and confirm our findings, we employed soft agar colony formation assays in conjunction with EGFR-TKI treatment. With the notion that SKBR3-RANK-c cells do not produce colonies in soft agar [10], we performed the assay only with MDA-MB-231-RANK-c and control cells treated with erlotinib or gefitinib (Figure 2B). Both control and MDA-MB-231-RANKc cells indicated a decreased ability for anchorage-independent growth in the presence of either erlotinib or gefitinib with no significant differences. However, MDA-MB-231-RANK-c cells seem to produce colonies of reduced size compared to control cells when treated with erlotinib or gefitinib, indicating that both agents in conjunction with RANK-c expression can reduce colony proliferation, but not the number of colony-initiating cells in the starting population of breast cancer cells (Figure 2C). Overall, RANK-c expression in combination with either erlotinib or gefitinib, depending on cell line, can inhibit proliferation and anchorage-independent growth of breast cancer cells.

### 3.3. TKIs Affects EGFR Downstream Signaling in RANK-C Expressing Breast Cancer Cells

**Figure 3:**
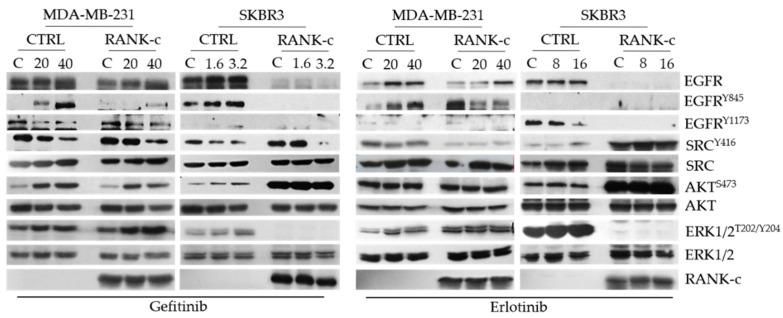
Immunoblots for the indicated proteins and phosphoproteins after treatment with the EGFR-TKIs gefitinib and erlotinib for 24 h (concentrations in μM) (C: untreated cells).

EGFR status and proteins that lie downstream of EGF-EGFR cascade were tested for activation by immunoblots for the relevant phosphorylated forms under erlotinib or gefitinib exposure. Phosphorylation of EGFR at tyrosine 845 and 1173 and activation of downstream signaling seems to be significantly deregulated in both ER-negative breast cancer cell lines (MDA-MB-231 and SKBR3) when treated with either gefitinib or erlotinib, in the presence of RANK-c (Figure 3). Furthermore, a significant deregulation of AKT and ERK1/2 pathways is observed in SKBR3-RANK-c cells that are already affected by the expression of RANK-c. Finally, ERK1/2 phosphorylation is not affected in MDA-MB-231 cells as a result of a mutant k-Ras protein that lies downstream of EGFR, in the specific cell line. In line with our observation of heterogeneity at the cellular level, the identified activation pattern presents also with heterogeneity at the molecular level concerning cell lines and treatments, indicating the complexity of pathway regulation in breast cancer cells upon treatment.

## 4. Discussion

In recent years survival rates for breast cancer patients present significant improvement. This is mostly due to the employment of massive mammogram screening programs for women but also further elucidating breast cancer biology that leads to development of better treatments [1]. Nevertheless, and especially in the case of ER-negative and triple-negative breast cancer, understanding disease biology has not provided the expected novel therapeutic tools [13,14]. It is characteristic that for triple-negative disease, chemotherapy and radiation therapy remain the standard of care for this group of patients while a plethora of targeted therapies (TKIs, monoclonal antibodies) have shown relatively low therapeutic efficacy levels [8,15].

Breast cancer is a highly heterogenous disease at both the molecular and histological levels. Still the only valid predictive biomarkers in everyday clinical practice are ER protein and HER2 receptor expression [16]. The identification of patients that could benefit from a specific therapeutic approach is of immense importance especially in the ER-negative and triple-negative disease settings [17]. The above can be tackled by the identification of novel predictive biomarkers for a smaller group of patients and by combining targeted therapies based on that biomarker expression profiles [18,19].

In previous work, we have identified RANK isoform c (RANK-c) as a regulator of stimuli-dependent NF-κB activation and an inhibitor of aggressiveness of ER-negative breast cancer. Furthermore, the characterized dependence of ER-negative breast cancer cells on NF-κB and the interaction of RANK-c with EGFR in breast cancer cell lines prompted us to further explore the possibility that RANK-c affects EGFR inhibition by known TKIs (erlotinib and gefitinib) [10,20]. With that in mind, we explored a publicly available dataset on breast cancer and identified that ER-negative, RANK-c positive samples presented with reduced phosphorylation of EGFR protein, corroborating our previous findings on RANK-c mode of action on EGFR. Furthermore, we treated ER-negative breast cancer cell lines stably expressing RANK-c with either erlotinib or gefitinib and observed a decrease in cell proliferation and colony size in soft agar depending on cell line and TKI employed, an observation previously reported for lapatinib [21]. At the same time, phosphorylation status of EGFR and downstream signaling molecules in RANK-c expressing cells indicated that several molecular pathways are deregulated after TKI treatments, presenting an additive effect on protein phosphorylation status that could explain functional cell properties observed for proliferation and colony formation.

These data indicate that RANK-c may serve as a possible marker of TKI responsiveness in ER-negative breast cancer, as with other possible protein biomarkers, but at the same time highlight the level of heterogeneity present in the two breast cancer cell lines, indicative of disease complexity [3,16,22]. Finally, while at the breast cancer cell line level RANK-c seems to predict response to erlotinib or gefitinib, the establishment of a relevant breast cancer mouse model to test the efficacy of TKIs in the presence of RANK-c protein remains an important goal, towards identifying RANK-c as a predictive biomarker.

## References

[1] Sung H., Ferlay J., Siegel R.L., Laversanne M., Soerjomataram I., Jemal A., Bray F. (2021). Global Cancer Statistics 2020: GLOBOCAN Estimates of Incidence and Mortality Worldwide for 36 Cancers in 185 Countries. CA Cancer J. Clin..

[2] Marchio C., Geyer F.C., Reis-Filho J.S., Loda M., Mucci L., Mittelstadt M., Van Hemelrijck M., Cotter M. (2017). Pathology and Molecular Pathology of Breast Cancer. Pathology and Epidemiology of Cancer.

[3] Lopez-Garcia M.A., Geyer F.C., Lacroix-Triki M., Marchio C., Reis-Filho J.S. (2010). Breast cancer precursors revisited: Molecular features and progression pathways. Histopathology.

[4] Marchio C., Reis-Filho J.S. (2008). Molecular diagnosis in breast cancer. Diagn. Mol. Pathol..

[5] Perou C.M., Sorlie T., Eisen M.B., van de Rijn M., Jeffrey S.S., Rees C.A., Pollack J.R., Ross D.T., Johnsen H., Akslen L.A. (2000). Molecular portraits of human breast tumours. Nature.

[6] Nakai K., Hung M.C., Yamaguchi H. (2016). A perspective on anti-EGFR therapies targeting triple-negative breast cancer. Am. J. Cancer Res..

[7] Merino Bonilla J.A., Torres Tabanera M., Ros Mendoza L.H. (2017). Breast cancer in the 21st century: From early detection to new therapies. Radiologia.

[8] Gupta G.K., Collier A.L., Lee D., Hoefer R.A., Zheleva V., Siewertsz van Reesema L.L., Tang-Tan A.M., Guye M.L., Chang D.Z., Winston J.S. (2020). Perspectives on Triple-Negative Breast Cancer: Current Treatment Strategies, Unmet Needs, and Potential Targets for Future Therapies. Cancers.

[9] Papanastasiou A.D., Sirinian C., Kalofonos H.P. (2012). Identification of novel human receptor activator of nuclear factor-kB isoforms generated through alternative splicing: Implications in breast cancer cell survival and migration. Breast Cancer Res..

[10] Sirinian C., Papanastasiou A.D., Schizas M., Spella M., Stathopoulos G.T., Repanti M., Zarkadis I.K., King T.A., Kalofonos H.P. (2018). RANK-c attenuates aggressive properties of ER-negative breast cancer by inhibiting NF-κB activation and EGFR signaling. Oncogene.

[11] Gao J., Aksoy B.A., Dogrusoz U., Dresdner G., Gross B., Sumer S.O., Sun Y., Jacobsen A., Sinha R., Larsson E. (2013). Integrative analysis of complex cancer genomics and clinical profiles using the cBioPortal. Sci. Signal..

[12] Cerami E., Gao J., Dogrusoz U., Gross B.E., Sumer S.O., Aksoy B.A., Jacobsen A., Byrne C.J., Heuer M.L., Larsson E. (2012). The cBio cancer genomics portal: An open platform for exploring multidimensional cancer genomics data. Cancer Discov..

[13] Sorlie T., Perou C.M., Tibshirani R., Aas T., Geisler S., Johnsen H., Hastie T., Eisen M.B., van de Rijn M., Jeffrey S.S. (2001). Gene expression patterns of breast carcinomas distinguish tumor subclasses with clinical implications. Proc. Natl. Acad. Sci. USA.

[14] Ades F., Zardavas D., Bozovic-Spasojevic I., Pugliano L., Fumagalli D., De Azambuja E., Viale G., Sotiriou C., Piccart M. (2014). Luminal B Breast Cancer: Molecular Characterization, Clinical Management, and Future Perspectives. J. Clin. Oncol..

[15] El Guerrab A., Bamdad M., Kwiatkowski F., Bignon Y.J., Penault-Llorca F., Aubel C. (2016). Anti-EGFR monoclonal antibodies and EGFR tyrosine kinase inhibitors as combination therapy for triple-negative breast cancer. Oncotarget.

[16] Martelotto L.G., Ng C.K., Piscuoglio S., Weigelt B., Reis-Filho J.S. (2014). Breast cancer intra-tumor heterogeneity. Breast Cancer Res..

[17] Freelander A., Brown L.J., Parker A., Segara D., Portman N., Lau B., Lim E. (2021). Molecular Biomarkers for Contemporary Therapies in Hormone Receptor-Positive Breast Cancer. Genes.

[18] McLaughlin R.P., He J., van der Noord V.E., Redel J., Foekens J.A., Martens J.W.M., Smid M., Zhang Y., van de Water B. (2019). A kinase inhibitor screen identifies a dual cdc7/CDK9 inhibitor to sensitise triple-negative breast cancer to EGFR-targeted therapy. Breast Cancer Res..

[19] Diluvio G., Del Gaudio F., Giuli M.V., Franciosa G., Giuliani E., Palermo R., Besharat Z.M., Pignataro M.G., Vacca A., d’Amati G. (2018). NOTCH3 inactivation increases triple negative breast cancer sensitivity to gefitinib by promoting EGFR tyrosine dephosphorylation and its intracellular arrest. Oncogenesis.

[20] House C.D., Grajales V., Ozaki M., Jordan E., Wubneh H., Kimble D.C., James J.M., Kim M.K., Annunziata C.M. (2018). IΚΚε cooperates with either MEK or non-canonical NF-kB driving growth of triple-negative breast cancer cells in different contexts. BMC Cancer.

[21] Abo-Zeid M.A.M., Abo-Elfadl M.T., Gamal-Eldeen A.M. (2019). Evaluation of lapatinib cytotoxicity and genotoxicity on MDA-MB-231 breast cancer cell line. Environ. Toxicol. Pharmacol..

[22] Bellizzi A., Greco M.R., Rubino R., Paradiso A., Forciniti S., Zeeberg K., Cardone R.A., Reshkin S.J. (2015). The scaffolding protein NHERF1 sensitizes EGFR-dependent tumor growth, motility and invadopodia function to gefitinib treatment in breast cancer cells. Int. J. Oncol..

